# Work Autonomous and Controlled Motivation on Chinese Employees' Work Performance and Innovative Work Behaviour: The Moderating Role of Financial Stress

**DOI:** 10.3389/fpsyg.2021.676063

**Published:** 2021-07-16

**Authors:** Feifei Ren, Qian Zhang, Xing Wei

**Affiliations:** Department of Psychology, Qingdao University, Qingdao, China

**Keywords:** autonomous motivation, controlled motivation, work performance, financial stress, innovative work behaviour

## Abstract

This study applied self-determination theory (SDT) to investigate the relationships between work motivation and work behaviour of Chinese employees and the moderating role of financial stress. Data were obtained from 245 employees of five organisations in China using a convenience sampling technique. The results indicated that autonomous motivation positively predicted work performance and innovative work behaviour, while controlled motivation had a positive effect only on work performance of employees. In addition, financial stress moderated the relationships between autonomous motivation and work performance and innovative work behaviour of employees. Specifically, the beneficial effect of autonomous motivation on work performance and innovative work behaviour disappeared when financial stress was high. The findings of the present study supported cultural similarities in the positive role of autonomous motivation and showed cultural differences in the role of controlled motivation. The implications of this study are also discussed.

## Introduction

Work motivation has been identified as an essential factor in predicting work behaviour of employees (e.g., Pinder, 1998; Zhang, 2019). Therefore, over the years, considerable research has examined how motivation of workers influences their work outcomes (e.g., Valero et al., 2015; Deci et al., 2017). While several theories have been proposed, one theory that has led to an impressive amount of research is self-determination theory (SDT; Ryan and Deci, 2000; Deci and Ryan, 2008).

In contrast to traditional theories that have treated motivation as a unitary concept that varies primarily in amount (e.g., Bandura, 1996; Baumeister and Vohs, 2007), SDT argued that there are different types of motivation and that the type of motivation is generally more important than the amount in predicting the important outcomes of life (Deci and Ryan, 2000). First, SDT divided motivation into intrinsic motivation and extrinsic motivation. Later, the conception of internalisation and types of regulation shifted the primary differentiation within SDT to a focus on autonomous versus controlled motivation. More specifically, SDT proposes that there are three types of internalisation (identified regulation, introjected regulation, and external regulation) that differ in the degree to which the regulations become integrated with a person's sense of self. Autonomous motivation comprises both intrinsic motivation and identified motivation in which people have identified with an activity's value and ideally will have integrated it into their sense of self (Gagné and Deci, 2005). Controlled motivation, in contrast, consists of both external regulation, in which one's behaviour is a function of external contingencies of reward or punishment, and introjected regulation, in which the regulation of action has been partially internalised and is energised by factors such as an approval motive, avoidance of shame, contingent self-esteem, and ego-involvements (Deci et al., 2017).

In recent years, there has been a dramatic increase in SDT-based research in the enterprise domain. Specifically, previous research has shown that autonomous motivation predicts less burnout and work exhaustion and is related to greater work satisfaction and performance (e.g., Gagné and Deci, 2005; Gillet et al., 2016; Olafsen and Bentzen, 2020). In contrast, controlled motivation has tended to show opposite results (Deci et al., 2017). Although studies on work motivation based on SDT have obtained many meaningful results, there are still several problems to be further explored.

First, prior research focused on the relationships between autonomous and controlled motivation and work outcomes has primarily been conducted in Western countries. Research into this issue is still rare in China. However, it is widely known that Chinese people's understanding and reaction to autonomy and control are different from those of Westerners (Markus and Kitayama, 2010). Therefore, the relations between autonomous and controlled motivation and work behaviour in Chinese societies might be different from those in Western countries. Researchers have argued that people in different cultures have strikingly different constructs of the self that can influence individual motivation. Specifically, a person in Western culture with an independent view of one's self should be motivated to engage in those actions that allow the expression of one's important self-defining, inner attributes (e.g., hardworking, caring, independent, and powerful), whereas a person in Eastern culture with an interdependent view of one's self should be motivated to engage in those actions that enhance or foster one's relatedness or connexion to others (Markus and Kitayama, 2010). To clarify the role of autonomous and controlled motivation in work behaviour of Chinese employees, the present study therefore examined the effect of autonomous and controlled motivation in Chinese culture.

In addition, research on organisations has paid much more attention to the main effect of autonomous and controlled motivation. Few studies have included potential variables moderating the relationship between motivation and work outcomes. However, previous studies found that the relationships between autonomous and controlled motivation and work outcomes varied as a function of contextual conditions (Gillet et al., 2016). One of the conditions was the pressure that employees experience. Previous research has found that financial stress affects work safety outcomes (Petitta et al., 2020), productivity, and interpersonal relationships in the workplace (Williams et al., 1996). Furthermore, in recent years, with burdens such as housing loan repayments, the implementation of the two-child policy, and increased expenditures and payments for children, financial stress has become an important source of stress (Long and Wang, 2015). Hence, the present study examined the moderating role of financial stress in relationships between autonomous and controlled motivation and work behaviour of Chinese employees.

**Figure 1:**
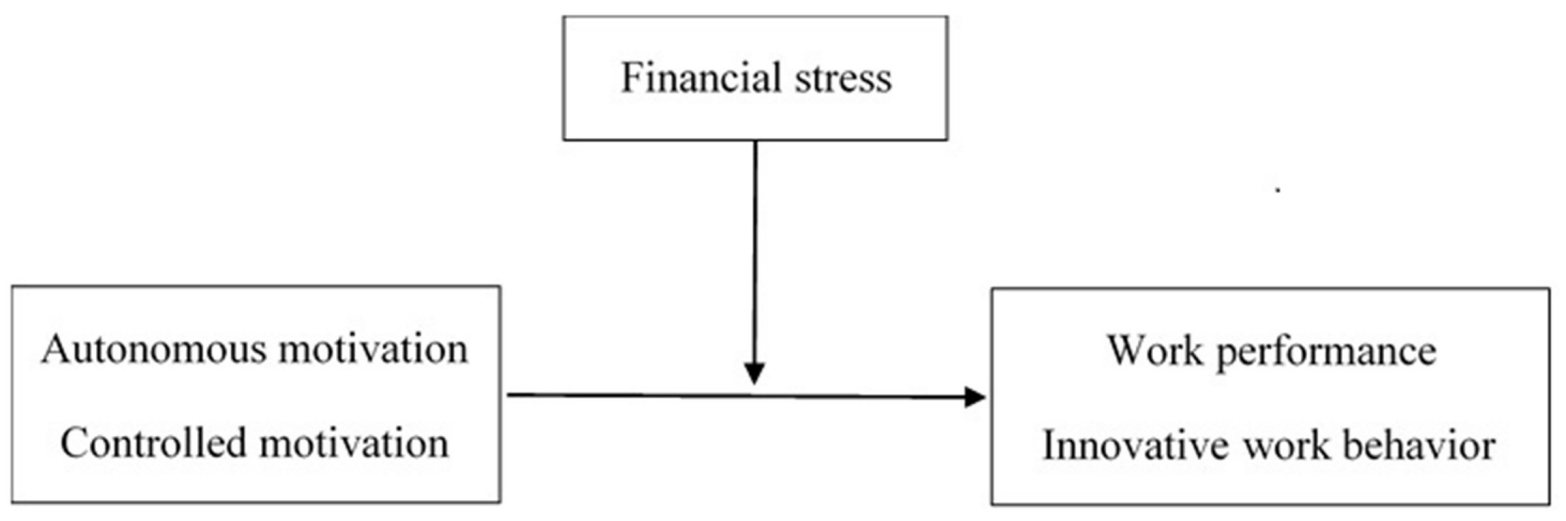
Conceptual model.

In summary, this study will examine the effects of autonomous and controlled motivation on work behaviour of Chinese employees and the moderating role of financial stress (Figure 1). In the present study, we mainly focused on two important work behaviours. The first behaviour is work performance, which refers to those types of behaviour explicitly required or expected by supervisors, coworkers, and role descriptions (Fay and Sonnentag, 2002). In addition to traditional work performance, we also consider innovative work behaviour, which refers to the production of ideas regarding products, practises, processes, or procedures that are novel and potentially useful to an organisation (Amabile, 1996). Research has shown that innovative work behaviour is becoming increasingly more important to enterprises. From the perspective of SDT, the fundamental driving force of innovative work behaviour comes from the individual's inner sense of self-determination and active consciousness.

The findings of the present study will provide evidence regarding the applicability of SDT-derived constructs to the Chinese cultural context. Furthermore, this moderating orientation should make a significant contribution to the motivation literature by showing that contextual factors may moderate the relationships of autonomous and controlled motivation and work behaviour. Furthermore, this perspective should be particularly relevant to understand and enrich motivational interventions.

### Effect of Autonomous and Controlled Motivation on Work Performance and Innovative Work Behaviour

Autonomous motivation is characterised by people engaging in an activity with a full sense of willingness, volition, and choice (Deci and Ryan, 2008). In contrast, with controlled motivation, the person engages in an activity due to experiencing pressure and demands to achieve specific outcomes that come from forces perceived to be external to the self (Deci and Ryan, 2008). In the work setting, autonomous motivation has been found to be associated with positive outcomes such as work engagement (e.g., Lopes and Chambel, 2017), job satisfaction (e.g., Gillet et al., 2016), and job effort (e.g., Gagné et al., 2015). This leads to positive outcomes because when autonomously motivated, employees perceive their work as congruent with their own values and interests. This representation allows the individual to fully partake in the activity. In contrast, controlled motivation has been generally associated with maladaptive work outcomes such as turnover intentions (e.g., Gillet et al., 2013), work anxiety (e.g., Gillet et al., 2016), and burnout (e.g., Gagné et al., 2015). Controlled motivation is associated with negative outcomes because individuals experience pressure to think, feel, or behave in particular ways, making goal pursuit less aligned with one's values and interests. Regarding the outcomes studied in the present study, Fernet et al. (2015) conducted research in two occupational settings (637 nurses and 210 school principals participated) and found that autonomous motivation was positively related to job performance, while controlled motivation was negatively associated with job performance. A longitudinal study with 116 employees conducted by Reizer et al. (2019) also proved the positive relation between autonomous motivation and job performance and the negative relation between controlled motivation and job performance. In addition, Erik (2019) reported that autonomous motivation was positively related to innovative work behaviour using a sample of 422 employees.

However, most of the above findings are obtained in the context of Western culture, and there is no direct empirical study on the relationships between autonomous and controlled motivation and work performance and innovative work behaviour in Chinese culture. However, the results regarding other work outcomes have suggested that the effect of autonomous motivation is universal regarding culture and the effect of controlled motivation is culture-specific to Chinese culture.

Specifically, Li et al. (2016) mentioned that autonomous motivation was positively associated with innovative work behaviour of teachers and promotion focus in a sample of 352 teachers in primary and middle schools in China. Zhang and Wu (2016) stated that autonomous motivation was positively related to proactive behaviour of employees using a sample of 529 employees from a Chinese enterprise. Nie et al. (2014) examined different predictive relations between work motivation and well-being in a sample of 266 teachers from two government middle schools in China. The study reported that autonomous motivation was positively related to job satisfaction. The reason that autonomous motivation has a beneficial impact on affect and behaviour in different cultural contexts may be that basic psychological needs (e.g., needs for autonomy) are universal (Chua et al., 2014). In contrast, studies examining the effects of controlled motivation have shown cultural differences. For instance, Zhang and Wu (2016) found that controlled motivation was positively related to proactive behaviour of employees. Nie et al. (2014) reported that introjected motivation, which is usually classified as controlled motivation, positively predicted job satisfaction after controlling for other types of motivation. Similarly, using a sample of 349 volunteers from Romania, a collective society such as China, Haivas et al. (2012) claimed that the correlations between introjected motivation and the satisfaction of all types of basic needs and work engagement were significantly positive. The reason for cultural-specific findings regarding controlled motivation might be that in an interdependent culture, Eastern people focused more on social, interpersonal, and relational styles (Markus and Kitayama, 2003). This type of person is likely to adjust to others by taking others' perspectives and reading others' expectations. Therefore, controlled motivation may have a positive effect in Chinese culture.

Base on the above reasoning, we hypothesised the following:

H1: Autonomous motivation was positively related to work performance and innovative work behaviour of Chinese employees.
H2: Controlled motivation was positively related to work performance and innovative work behaviour of Chinese employees.

### Moderating Role of Financial Stress

Financial stress is defined as an individual's perception of financial inadequacy and her/his financial concerns and worries (Mills et al., 1992). Previous research has found that financial stress is related to employees' behaviour and psychological status. For example, Mills et al. (1992) interviewed 330 adults face-to-face. They found that economic strain has a detrimental effect on both employed men's and women's psychological well-being. Petitta et al. (2020) conducted a cross-country research using data from 498 employees in the United States and 366 in Italy and found that financial stress exerted a positive effect on workplace injuries and accident under-reporting. Harris Allen (2008) analysed the data provided by the HRA's (Human Resource Audit) developer using structural equation techniques. Using data from 17,821 respondents, the study found that financial concerns contributed to presenteeism and absenteeism at work of employees.

Lazarus's transactional model of stress indicated that appraisal and coping occurred when encountering stress (e.g., Lazarus and Folkman, 1987). If the environment is appraised as taxing or exceeding the person's resources and endangering his or her well-being, coping is activated. If the encounter is successfully resolved, coping ceases and positive outcome results. Otherwise, negative affect and physiological disturbances persist, ultimately damaging adaptational outcomes (i.e., psychological well-being, somatic health, and social functioning). Accordingly, when financial stress is low, individuals are more likely to use resources as a coping mechanism or stress-reducing action. Research has suggested that work motivation is an important personal resource for individuals (e.g., Gagné and Deci, 2005). Therefore, a low level of financial stress might lead autonomous and controlled motivation to have more influence on work outcomes. However, the coping hypothesis might not be suitable for high-level financial stress. When financial stress is too high, employees might believe that no reasonable level of effort will be adequate to alter the troubling situation. The situation might be out of employees' control, and they will tend to have low motivation to expend effort on coping. Therefore, a high level of financial stress might hinder the beneficial effect of autonomous and controlled motivation on work performance and innovative work behaviour of employees.

Base on the above reasoning, we hypothesised the following:

H3: High level of financial stress will weaken the positive relationship between autonomous motivation and work performance and innovative work behaviour.
H4: High level of financial stress will weaken the positive relationship between controlled motivation and work performance and innovative work behaviour.

## Materials and Methods

### Participants and Procedures

The study used convenience sampling technique. To improve the diversity, fairness, and inclusiveness of the data, data were collected from the employees of five organisations engaged in different industries in Qingdao City, Shandong Province, eastern China. The organisations included one information technology company, one rubber service-oriented company, one robotics company, one property management company, and one investment management firm.

Three hundred and nineteen employees were asked to complete a self-reported questionnaire at the workplace during company time. After deleting invalid questionnaires, there were 245 valid subjects. Of the sample, 139 were men and 106 were women. A total of 45.7% were in the 20–29 age group, and 58.4% were in the 30–40 age group. For education, 15.5% had a master's degree or higher, 51% had a bachelor's degree, 22.9% had an associate degree, and 10.6% had a high school degree. Respondents averaged 7.07 years (*SD* = 4.58) of organisational tenure.

To ensure proper translation, the translation work followed the International Test Commission (Hambleton, 1994). The questionnaire was first translated into Chinese and then translated back into English. Before issuing questionnaires, the questionnaire was first reviewed and confirmed by the directors of the human resource departments of the organisations.

### Measures

#### Autonomous and Controlled Motivation

Autonomous and controlled work motivation were assessed with the Multidimensional Work Motivation Scale (MWMS) developed by Gagné et al. (2015). The total scale consists of 16 items. The participants' task was to assess the level of statements' conformity with their beliefs regarding why they are or if they would put effort into their current job on a 7-point scale (1 = *not at all* to 7 = *completely*). The scale includes six subscales: intrinsic motivation (three items; e.g., “because I have fun doing my job”), identified regulation (three items; e.g., “because putting effort into this job aligns with my personal values”), introjected regulation (four items; e.g., “because I have to prove to myself that I can”), extrinsic regulation-material (three items; e.g., “because others will reward me financially only if I put enough effort in my job”), and extrinsic regulation-social (three items; e.g., “to get others' approval (e.g., supervisor, colleagues, family, clients…)”). The first two subscales were added and averaged to estimate autonomous motivation, and the other subscales were added and averaged to estimate controlled motivation. The Cronbach's α was 0.94 for autonomous motivation and 0.92 for controlled motivation.

#### Work Performance

Work performance was measured by a scale taken from Farh and Cheng (1997). The scale consists of four items (e.g., makes a significant contribution to the overall performance of our work unit). Participants responded to items on a 5-point Likert scale anchored by 1 (*not true at all*) and 5 (*very true*). The Cronbach's α for this scale was 0.61.

#### Innovative Work Behaviour

Following the guidelines of Baer and Oldham (2006), we assessed each employee's creativity using four items derived from those developed by Zhou and George (2001). The sample items included the followings: suggests many creative ideas that might improve working conditions at the organisation and often comes up with creative solutions to problems at work. The items were rated on a scale that ranged from *not at all characteristic* (1) to *very characteristic* (5). The Cronbach's α for this scale was 0.83.

#### Financial Stress

The financial stress scales for this study were developed and adopted based on different studies (Ma et al., 2014; Yang, 2017). The sample items included the following: comparing my position and salary with others makes me feel stressed and my income cannot meet my monthly living expenses. The responses were coded on a 5-point Likert-type scale ranging from 5 = *very characteristic* to 1 = *not at all characteristic*. The composite measure had an acceptable level of reliability as measured by a Cronbach's alpha of 0.82.

#### Control Variables

According to previous studies, age, gender, education, and organisation tenure have been shown to explain variance in work performance and innovative work behaviour (e.g., Lei et al., 2015; Ye et al., 2015; Zhang and Long, 2016). Therefore, the effects of gender, age, education, and organisation tenure were statistically controlled to elicit their potential confounding effects.

#### Common Method Biases

The reliance on self-reported questionnaire data may possibly cause common method variance. In this study, we reduced the concern by protecting the anonymity of the respondents and including some reverse-coded items in our questionnaire. In addition, we used Harman's one-factor test to address the common method bias issue. The results of this analysis showed that eight factors with eigenvalues greater than 1.0 emerged, and the first factor accounted for only 26.5% of the total variance. These cheques suggested that common method variance was not a serious concern in this study (Podsakoff and Organ, 1986).

## Results

Table 1 presents the descriptive statistics, reliabilities, and correlations for all measures. Autonomous motivation showed significant positive correlations with work performance and innovative work behaviour (*r* = 0.28, *p* < 0.001; *r* = 0.19, *p* = 0.004, respectively). Controlled motivation was significantly and positively related to work performance (*r* = 0.17, *p* = 0.007), whereas it was positively but nonsignificantly related to innovative work behaviour (*r* = 0.71, *p* > 0.05). Financial stress showed significant negative correlations with innovative work behaviour (*r* = −0.15, *p* = 0.019) and was positively but nonsignificantly related to work performance (*r* = 0.01, *p* > 0.05).

**Table 1:** Means, standard deviations, correlations, and Cronbach's α for the main study variables.

**Variables**	***M***	***SD***	**1**	**2**	**3**	**4**	**5**
1. AM	4.87	1.12	(0.94)				
2. CM	4.50	1.08	0.76^***^	(0.92)			
3. WP	3.67	0.52	0.28^***^	0.17^**^	(0.61)		
4. IWB	3.34	0.71	0.19^**^	0.08	0.51^***^	(0.83)	
5. FS	3.52	0.79	−0.07	0.01	0.01	−0.15^*^	(0.82)

*N, 245; Reliabilities are in parentheses. AM, autonomous motivation; CM, controlled motivation; WP, work performance; IWB, innovative work behaviour; FS, financial stress*.

* *p < 0.05*,

** *p < 0.01*,

*** *p < 0.001*.

Table 2 presents the direct effects of autonomous motivation and controlled motivation on work performance and innovative work behaviour. The results showed that autonomous motivation positively and significantly predicted work performance (β = 0.27, *p* < 0.001) and innovative work behaviour (β = 0.13, *p* = 0.046), which explained 7 and 2% of the variance, respectively. Controlled motivation showed a significantly positive correlation with work performance (β = 0.16, *p* = 0.025), which explained 2% of the variance. However, the relationship between controlled motivation and innovative work behaviour was not significant (β = 0.04, *p* > 0.05).

**Table 2:** Regression analysis of dependent variables on autonomous motivation and controlled motivation.

**Variable**		**Work performance**	**Innovative work behaviour**
Autonomous motivation	β	0.27^***^	0.16^*^
	*R^2^*	0.07^***^	0.04^*^
	Δ*R*^2^	0.07^***^	0.03^*^
Controlled motivation	β	0.13^*^	0.04
	*R^2^*	0.10^*^	0.09
	Δ*R*^2^	0.02^*^	0.00

*As shown in Table 1, there was a strong positive correlation between autonomous motivation and controlled motivation (r = 0.76, p < 0.001). In order to avoid possible suppression effects, we separately examined the effects of autonomous and controlled motivation after controlling the influence of demographic variables: age, gender, education, and tenure in the regression analyses*.

*Standardised beta coefficients are shown*.

* *p < 0.05*,

*** *p < 0.001*.

To reduce the multicollinearity problem in the interaction terms, the variables were first centred, as recommended by Aiken and West (1991). Then, the control variables were entered in Step 1, followed by testing the main effects of motivation and financial stress in Step 2. The interaction term was added in Step 3 to provide a test of the moderating effects. In Table 3, the hierarchical moderated regression analysis table for only AM × FS on WP as well as AM × FS on IWB showed that the interaction terms for autonomous motivation and financial stress on work performance and innovative work behaviour were significant (β = −0.17, *p* = 0.009; β = −0.16, *p* = 0.044, respectively), which explained 3 and 2% of the variance, respectively. This result indicated that financial stress moderated the relationship between autonomous motivation and work performance and innovative work behaviour. In Table 4, the hierarchical moderated regression analysis table for only CM x FS on WP showed that the interaction terms for controlled motivation and financial stress on work performance were nonsignificant (β = −0.268, *p* > 0.05), indicating that the relationship between controlled motivation and work performance was not moderated by financial stress. In addition, because the results of the regression analysis of innovative work behaviour on controlled motivation were not significant, hierarchical regression analysis for the effect of financial stress on the relationship between controlled motivation and innovative work behaviour was not conducted.

**Table 3:** Hierarchical regression analyses to assess the effect of financial stress (FS) in affecting the relationships between autonomous motivation (AM), work performance (WP), and innovative work behaviour (IWB).

	**Work performance**			**Innovative work behaviour**		
**Variable**	***β***	***R***^**2**^	**Δ*****R***^**2**^	***β***	***R***^**2**^	**Δ*****R***^**2**^
Step 1		0.02	0.02		0.08^***^	0.08^***^
Age	0.05			0.11		
Gender	−0.00			−0.10		
Education	−0.01			0.11		
Tenure	0.10			0.18		
Step 2		0.09^***^	0.07^***^		0.11	0.02
AM	0.27^***^			0.13^*^		
FS	0.04			−0.09		
Step 3		0.12^**^	0.03^**^		0.12^*^	0.02^*^
AM × FS	−0.16^**^			−0.13^*^		

*AM, autonomous motivation; FS, financial stress*.

*Standardised beta coefficients are shown*.

* *p < 0.05*,

** *p < 0.01*,

*** *p < 0.001*.

**Table 4:** Hierarchical regression analyses to assess the effect of financial stress (FS) in affecting the relationship between controlled motivation (CM) and work performance (WP).

	**Work performance**		
**Variable**	**β**	***R***^**2**^	**Δ*****R***^**2**^
Step 1		0.02	0
Age	0.06		
Gender	0.03		
Education	0		
Tenure	0.08		
Step 2		0.05	0.02
CM	0.17^*^		
FS	0.02		
Step 3		0.05	0.03
CM × FS	−0.27		

*CM, controlled motivation; FS, financial stress*.

*Standardised beta coefficients are shown*.

* *p < 0.05*.

**Figure 2:**
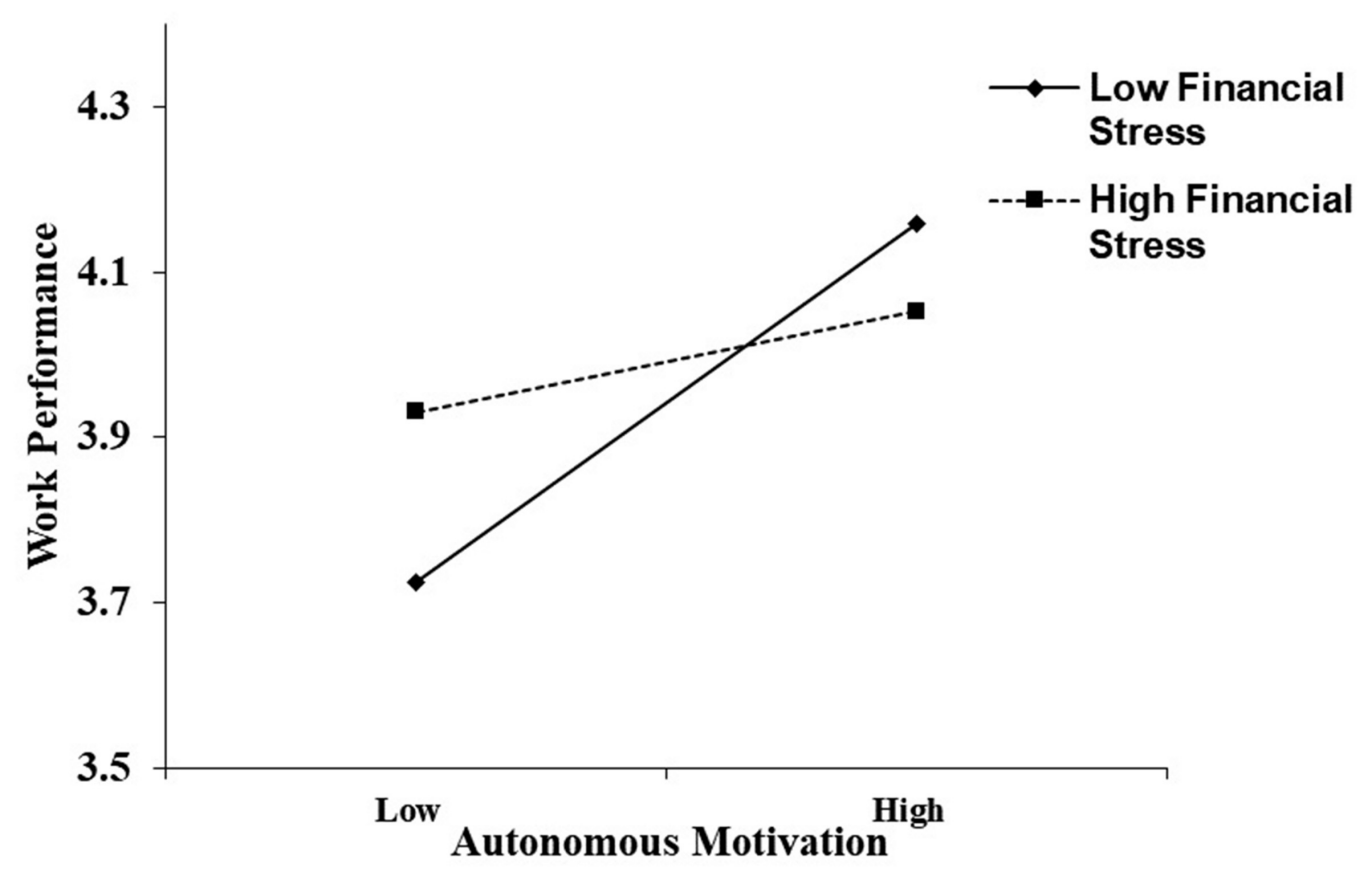
Interaction effect of autonomous motivation and financial stress on work performance. Conditional regressions of work performance on autonomous motivation were conducted when financial stress was high (*M*+1 *SD*) and low (*M*−1 *SD*). Endpoints of the lines represented the scores of work performance when autonomous motivation was low or high.

**Figure 3:**
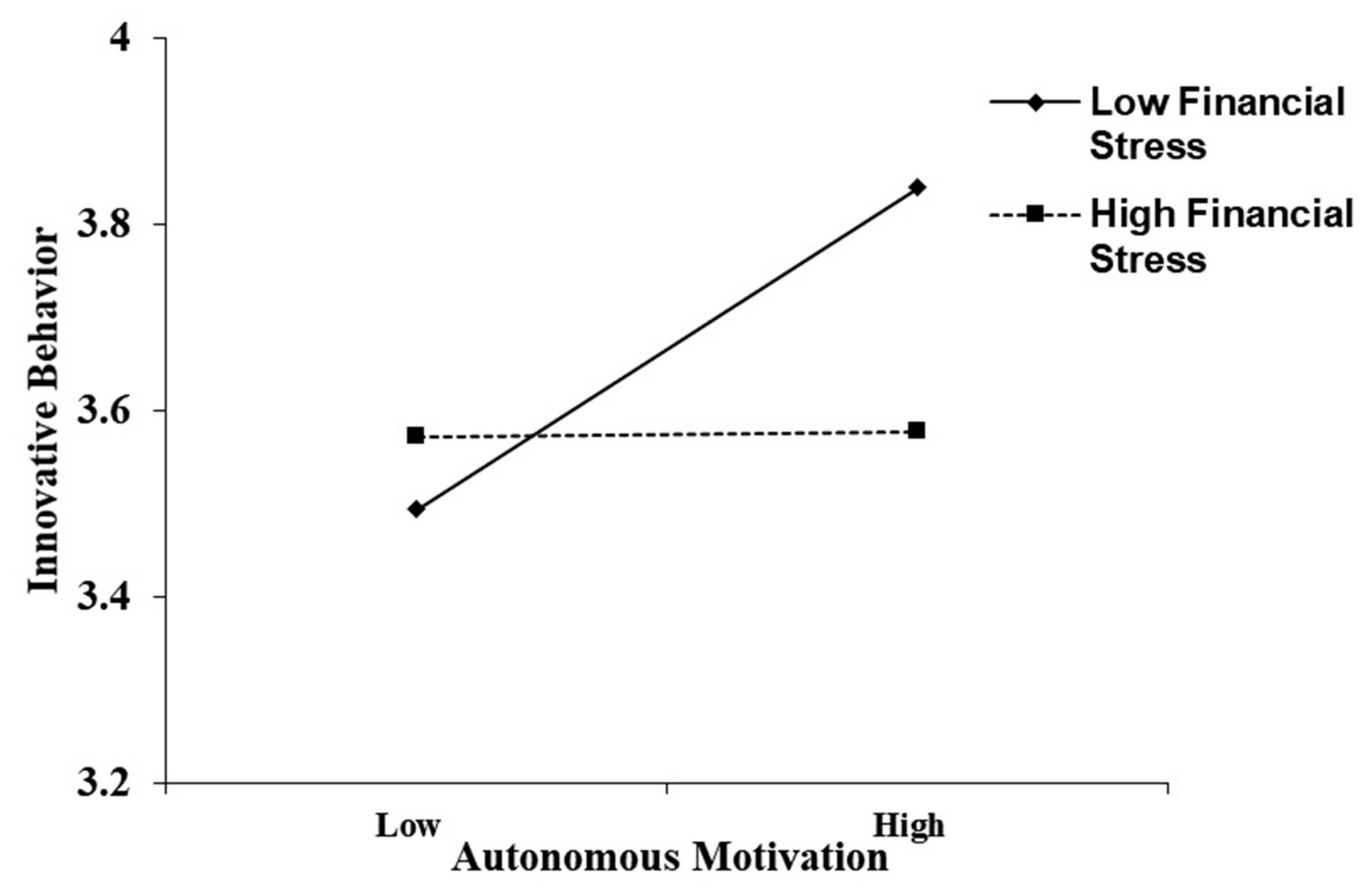
Interaction effect of autonomous motivation and financial stress on innovative work behaviour. Conditional regressions of innovative work behaviour on autonomous motivation were conducted when financial stress was high (*M*+1 *SD*) and low (*M*−1 *SD*). Endpoints of the lines represented the scores of innovative work behaviour when autonomous motivation was low or high.

Simple slope tests were then conducted to analyze the moderating effects of financial stress on the relationships between autonomous motivation and work performance and innovative work behaviour. As suggested by Aiken and West (1991), simple slopes were estimated at two levels: low (one SD below the mean of financial stress) and high (one SD above the mean of financial stress). As shown in Figure 2, autonomous motivation was positively related to work performance when financial stress was low (simple slope = 0.41, *t* = 4.97, *p* < 0.001), whereas autonomous motivation was not related to work performance when financial stress was high (simple slope = 0.13, *t* = 1.56, *p* = 0.12). As shown in Figure 3, autonomous motivation was positively related to innovative work behaviour when financial stress was low (simple slope = 0.24, *t* = 2.87, *p* = 0.005), whereas autonomous motivation was not related to innovative work behaviour when financial stress was high (simple slope = 0.02, *t* = 0.25, *p* = 0.81).

## Discussion

The relationships between work motivation and work behaviour of employees have long been a focus in work organisations. The present study contributed to the existing literature by examining the relationships between autonomous and controlled motivation and work performance and innovative work behaviour of Chinese employees based on SDT's framework of motivation and the moderating role of financial stress.

### Effect of Autonomous and Controlled Motivation on Chinese Employees' Work Performance and Innovative Work Behaviour

The present study found that autonomous motivation had a significant positive relationship with work performance and innovative work behaviour of Chinese employees. The results agreed with previous findings in Western settings that autonomous motivation was related to more positive affect and enhanced performance, particularly heuristic activities and more creativity (Deci and Ryan, 2008). These findings confirmed that autonomous motivation appeared to be universally important and played a significant role for Chinese employees in achieving positive work outcomes. As for controlled motivation, the results demonstrated that it was significantly and positively correlated with work performance of employees. This result was inconsistent with findings of Western societies that controlled motivation was negatively related to performance (Gagné and Deci, 2005; Deci et al., 2017). Eastern people behave in an interdependent manner, focusing more on social, interpersonal, and relational styles (Markus and Kitayama, 2003). This type of person is likely to adjust to others, take others' perspectives, and read others' expectations. Such tendencies could manifest by acknowledging one's obligations or roles in a specific situation and an awareness of others' larger role in influencing who they are and what they should do (Markus and Kitayama, 2010). Therefore, external pressure and regulation might be motivational for people in an Eastern culture. These might be the causes of cultural differences in terms of the effects of controlled motivation on work performance of employees.

However, it is noteworthy that controlled motivation did not show a positive effect on innovative work behaviour of Chinese employees. This is likely because innovative work behaviour mainly assesses performance in heuristic and complex tasks. Previous studies found that controlled motivation might have a short-term performance advantage for mundane and boring tasks but detracts from performance on tasks that require cognitive flexibility, creativity, or deep information processing (Gagné and Deci, 2005). These results suggested that even in the context of Chinese culture, in which people have a higher tolerance of control than Western countries, controlled motivation cannot promote innovative performance.

### Moderating Role of Financial Stress

Another significant finding in this study was that financial stress played a moderating role in the relationships between autonomous motivation and positive work outcomes. The results indicated that individual characteristics and environmental influences interactively contribute to work behaviour of employees. Specifically, autonomous motivation was more strongly related to positive work outcomes with low-level financial stress, and the positive relations became non-significant under high-level financial stress. The results were in line with Gillet et al's. (2016) finding that the effect of autonomous motivation on satisfaction varied as a function of role ambiguity. This result is supported by previous research stating that high-level financial stress could operate as a hindrance stressor and lead to negative emotions and a passive coping style (Wallace et al., 2009). With a passive coping style, individuals tend to decrease effort by retreating from the situation or engaging in destructive behaviours to make individuals feel better (Spector, 2002; Wallace et al., 2009).

However, financial stress did not moderate the relationship between controlled motivation and work performance. The possible reason might be that people with controlled motivation are better at handling external pressure. Therefore, high financial stress did not influence the effect of controlled motivation on work performance.

### Limitations and Future Research

In interpreting the findings of this study, some limitations must be considered. First, this study used a cross-sectional design to explore the relationship between autonomous and controlled motivation and work outcomes. Because of the cross-sectional nature of the data, there may be doubts regarding the direction of the causality. Hypotheses were based on the logic that autonomous motivation would influence affect and work outcomes of employees. However, it is also possible that employees with better performance and more innovative work behaviour have a tendency to work with volition and a sense of choice (i.e., autonomous motivation) or with pressure and external demand (i.e., controlled motivation). Thus, longitudinal research is needed to examine the causal relationship between variables. Second, all data gathered were self-reported. In future research, this limitation might be avoided by evaluating these variables objectively rather than subjectively. Third, this study found that controlled motivation contributed to better work performance. This finding might be interpreted according to the different understanding and reaction to control between Chinese and Westerners, which requires further empirical study for verification.

### Implications and Conclusion

In addition to providing direct evidence regarding the applicability of SDT-derived constructs to the Chinese working context, this study also has some implications for practical management. First, our findings suggested that autonomous motivation was conducive to work performance and innovative work behaviour of Chinese employees. Therefore, to achieve organisational effectiveness and success, organisations should provide an autonomy-supportive climate to enhance autonomous motivation of employees. Although the present study found that controlled motivation could also promote work performance of Chinese employees, we should be cautious about exhibiting controlling interpersonal behaviours as controlled motivation cannot promote innovative work behaviour of employees; moreover, the beneficial effect on work performance is smaller than that of autonomous motivation. In addition, our study found that the positive effect of autonomous motivation totally disappeared when employees perceived a high level of financial stress. Therefore, instead of only promoting autonomous motivation, organisations should also pay attention to contextual factors that might influence the effect of motivation. For example, offering better pay appropriately to reduce financial stress of employees might enhance the beneficial effects of autonomous motivation on work performance and innovative work behaviour.

In conclusion, the present study expanded our knowledge of the roles of autonomous and controlled motivation in work behaviour of Chinese employees. That is, autonomous motivation contributed to better work performance and innovative work behaviour, and controlled motivation contributed to better work performance but had no significant relationship with innovative work behaviour. These findings suggested that autonomous and controlled motivation were significant predictors of work behaviour of Chinese employees. In addition, these findings supported cultural similarities in the effects of autonomous motivation and suggested cultural differences in the effect of controlled motivation. Moreover, the innovativeness of work behaviour affects the effect of controlled motivation. Finally, the present study found a moderating role of financial stress between autonomous motivation and work behaviour of employees. This finding suggested that attention should be given to other variables that may influence work motivation effectiveness in organisational settings.

## Data Availability Statement

The raw data supporting the conclusions of this article will be made available by the authors, without undue reservation.

## Ethics Statement

The studies involving human participants were reviewed and approved by Experimental Ethics Committee of the Department of Psychology of Qingdao University. The patients/participants provided their written informed consent to participate in this study.

## Author Contributions

FFR, QZ, and XW contributed to conception and design of the study. FFR and QZ organised the database, performed the statistical analysis, and wrote the first draft of the manuscript. XW was the holder of the research project, assisted statistical analyses, and participated in developing the instruments and coordination. All authors contributed to the manuscript revision and read and approved the submitted version.

## Conflict of Interest

The authors declare that the research was conducted in the absence of any commercial or financial relationships that could be construed as a potential conflict of interest.
